# Development and Validation of a ^18^F-Flortaucipir PET Visual Stratification Method

**DOI:** 10.2967/jnumed.124.268700

**Published:** 2025-04

**Authors:** Ilke Tunali, Jian Wang, Anupa K. Arora, Min Jung Kim, Sergey Shcherbinin, Michael Pontecorvo, Leonardo Iaccarino

**Affiliations:** 1 Eli Lilly and Company, Indianapolis, Indiana; and; 2 Eli Lilly Italia S.p.A, Sesto Fiorentino, Italy

**Keywords:** Alzheimer disease, flortaucipir PET, visual read method, high tau

## Abstract

Tau PET quantitation methods have been used in research settings and clinical trials to measure tau burden for diagnostic, staging, and prognostic purposes. However, these methods require specialized software, skilled analysts, and advanced image processing. We developed a novel ^18^F-flortaucipir PET (FTP, or Tauvid) visual read method enabling stratification of patients with Alzheimer disease (AD) according to the tau level without the need for quantitation. An independent reader study (I7E-AV-A26) was conducted to test this method against a quantitation-based high-tau standard of truth. **Methods:** A total of 140 baseline or screening FTP scans were randomly selected from the TRAILBLAZER-ALZ 2 phase 3 trial (NCT04437511). Five qualified imaging physicians were trained for the FTP visual stratification method, using previously identified thresholds and cortical regions of interest thought to optimally stratify high-tau and non–high-tau scans. Positive and negative percent agreement (PPA and NPA, respectively) between visual stratifications and quantitation-based high tau (AD-signature SUV ratio > 1.46) were calculated. Predefined success criteria were met if the lower bounds of a 2-sided 95% CI for PPA and NPA were 50% or greater for at least 3 of the 5 readers. Inter- and intrareader reliability were assessed using Fleiss κ (*n* = 140) and Cohen κ (*n* = 20 test–retest scans) metrics. **Results:** The median PPA and NPA were 83.4% and 88.9%, respectively, with lower bounds of 2-sided 95% CIs being 50% or greater for all readers. The Fleiss κ-point estimate was 0.8882 (95% CI, 0.8356–0.9409) and the Cohen κ-point estimate was 0.9599 (95% CI, 0.9049–1.000) for all readers, indicating almost perfect inter- and intrareader agreement. Study I7E-AV-A26 successfully validated the feasibility of the FTP visual stratification method, possibly supporting AD staging and prognosis with high inter- and intrareader agreements, confirming the reliability of the method. **Conclusion:** Future investigations may include expanding the validation dataset, including real-world clinical data from diverse populations, using autopsy confirmation, exploring alternative regions and thresholds for other tau PET stratifications, and assessing differences in treatment response among visually stratified participants enrolled in disease-modifying therapy trials.

## Visual Abstract

**Figure:**
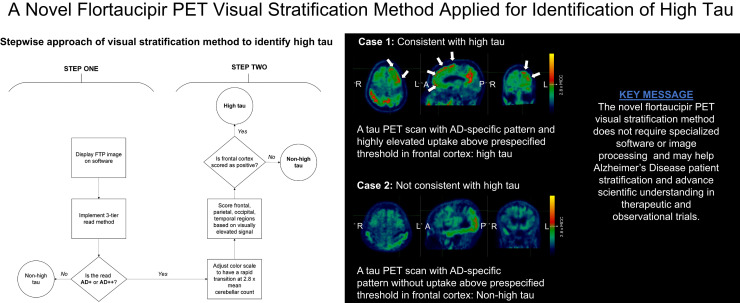

Alzheimer disease (AD) is a chronic neurodegenerative disease pathologically characterized by the accumulation of amyloid-β plaques and neurofibrillary tau tangles (NFTs) in the brain (1). PET is a critical imaging tool and is considered the gold standard for in vivo diagnosis and staging of AD through quantitation and visual interpretation methods (2–5). Multiple PET tracers have been developed in the last decade that can estimate the density and distribution of NFTs by selectively binding to aggregated tau or to assess amyloid pathology by binding to amyloid-β plaques (6).

^18^F-flortaucipir (FTP, or TAUVID [Lilly]) is currently the only tau imaging agent approved by the U.S. Food and Drug Administration (7) and the European Medicines Agency (8) that can be used to estimate the density and spatial distribution of aggregated NFTs in the brain (2). In the seminal PET-to-autopsy study, positive visual interpretation of FTP signal patterns was shown to associate with an advanced Braak NFT stage observed at autopsy, supporting the neuropathologic diagnosis of AD (9). Cross-sectional studies have demonstrated a strong correlation between both quantitatively (10) and visually assessed (11) tau PET signal and cognitive status in AD patients. Longitudinal studies have shown that increased global or regional tau PET signals captured visually (11) or quantitatively (10,12) were associated with more rapid cognitive and functional decline, informing AD prognosis. In one of those studies (12), patients were split into quartiles (e.g., no tau, low tau, medium tau, and high tau) based on their FTP signal measured using SUV ratio (SUVr). The cognitive decline measures worsened progressively from the no-tau group to the high-tau group.

In research settings and clinical trials, quantitation methods (13–17) have been used primarily to measure tau burden. However, this approach presents challenges, as it requires specialized software and advanced image processing steps, which can be time consuming. On the other hand, visual read methods can be more practical in clinical settings, as they can be performed by trained imaging physicians using widely available software, allowing for timely execution. The U.S. Food and Drug Administration and European Medicines Agency labels for FTP provide a visual read interpretation method that allows the identification of widely distributed tau neuropathology (Braak V/VI, B3) with a visual interpretation of a positive FTP scan. An investigational 3-tier FTP visual read method further differentiates positive scans based on regional patterns and stratifies by moderate and advanced tau AD patterns (τAD+ and τAD++, respectively) (9). However, these visual read approaches are not able to differentiate positive FTP scans with varying levels of quantitation-based tau PET groups (e.g., medium tau vs. high tau among positive τAD++ pattern scans) (5,6). We developed (18) a visual read stratification method that can classify AD patients based on different quantitation-based tau levels. The method is a natural extension to the 3-tier investigational FTP visual read method that was previously developed (12) and used in clinical trials for screening patients (13,14).

In this article, we present the findings of an independent reader study (I7E-AV-A26; hereafter study A26) that aimed to validate the feasibility of a novel tau PET visual stratification method and its ability to stratify patients by high tau and non–high tau using a subset of screening or baseline FTP scans acquired in a clinical trial.

## MATERIALS AND METHODS

### Image Data

**FIGURE 1.**
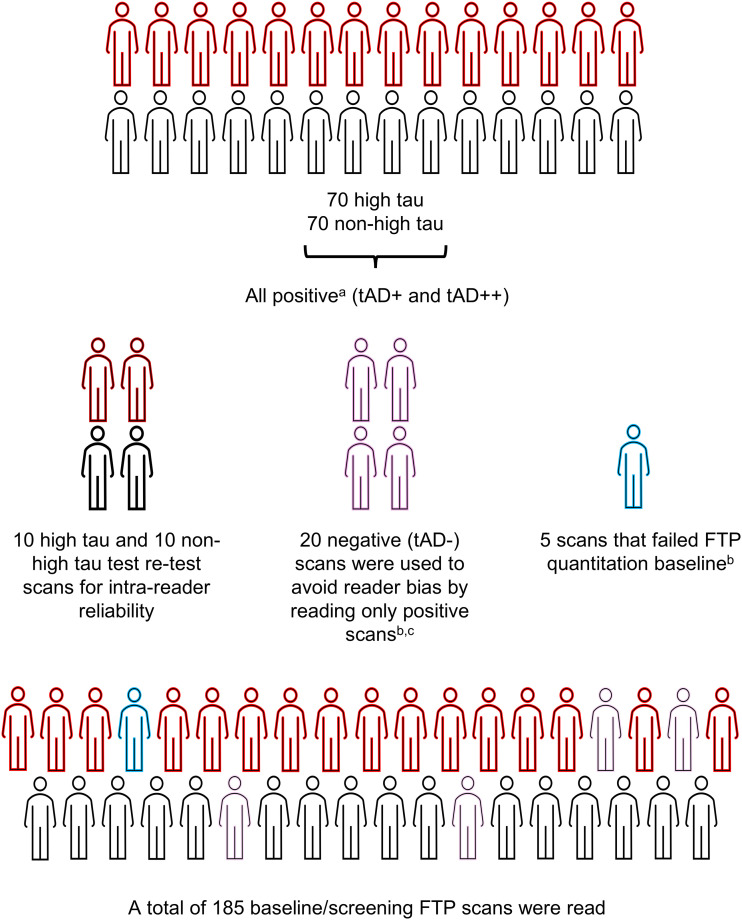
Schema of study A26. ^a^Proportion of scans with τAD+ (∼5%) and τAD++ (∼95%) reflected prevalence in TB2 study. ^b^Scans that were negative or had failed FTP quantitation were not used for calculating endpoints. ^c^Participants with negative scans were identified from screen-failed subjects to TB2 study.

Study A26 included 185 screening or baseline scans from the TB2 trial (TRAILBLAZER-ALZ2; NCT04437511) (Fig. 1). The image data used in this study were selected from screening or baseline FTP scans that were acquired as part of that trial. The TB2 trial is a randomized, double-blind, placebo-controlled phase 3 trial to evaluate the safety and efficacy of donanemab in participants with early symptomatic AD (prodromal AD and mild dementia due to AD) with the presence of brain amyloid and tau pathology (12). Quantitation of FTP PET was performed using an AD-signature weighted neocortical region with respect to a PERSI reference region (also referred to as MUBADA-PERSI SUVr (10,19)). This target region was developed as a global neocortical region weighted at the voxel level, with posterior areas, including temporal, parietal, and occipital, carrying higher weights overall (19). The TB2 study participants with an SUVr of less than 1.10 (3) were excluded from the trial, except for participants with a tau PET topographic deposition pattern consistent with τAD++, who were included regardless of their AD-signature weighted neocortical SUVr. An SUVr cut point of 1.46 (3) was used to identify participants with high tau (SUVr > 1.46). All other participants (SUVr ≤ 1.46) were classified as low or medium tau.

Of the 185 scans, 140 were randomly selected from scans that were visually read as τAD+ or τAD++ during the screening period of the TB2 trial and were randomized. An effort was made to balance the number of quantitation-based high-tau and non–high-tau scans (70 high tau and 70 non–high tau). Additionally, 20 randomly selected scans were used as test–retest scans for assessing intrareader reliability. These scans were selected from the original 140 τAD+ or τAD++ scans and were evenly balanced between the number of quantitation-based high-tau and non–high-tau scans. Twenty τAD− scans were randomly selected from subjects who were not randomized to TB2 to avoid potential reader bias due to reading only scans that are consistent with AD (τAD+ or τAD++). Finally, a set of 5 scans that failed initial quantitation at screening was included to assess the feasibility of the proposed visual read method when quantitation is difficult to perform.

### FTP PET Visual Stratification Method

The FTP PET visual interpretation method used in this study is a natural extension to the 3-tier investigational FTP visual read method that was previously defined (9). Readers using the 3-tier visual read method first estimate the mean counts in the cerebellar region (mean cerebellar count [MCC]) and then adjust the color scale to set a visual threshold to discriminate voxels with increased signal (defined as >1.65 times the MCC) compared with the background. Once the color scale is adjusted, readers examine 6 specified brain regions bilaterally (lateral anterior temporal, lateral posterior temporal, occipital, parietal, precuneus, and frontal lobes) and score each region as either positive or negative based on the presence of an elevated signal. Scans are evaluated using predefined rules (9) that assess the regional patterns of tracer uptake and are considered either not consistent with AD (τAD− pattern) or consistent with AD (τAD+ or τAD++). Further details of the 3-tier investigational read method are in the supplemental materials (available at http://jnm.snmjournals.org) (20).

**FIGURE 2.**
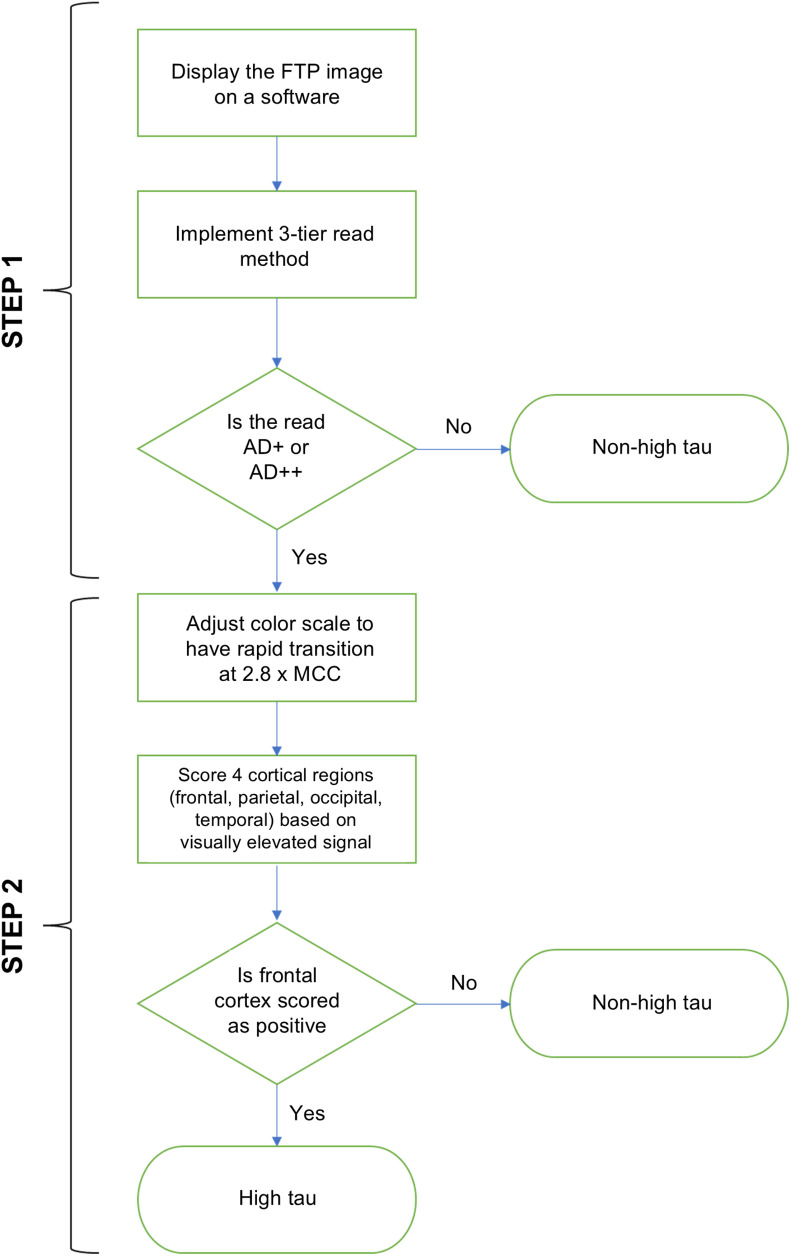
Algorithm displays stepwise approach of visual stratification method applied to identify high tau.

**FIGURE 3.**
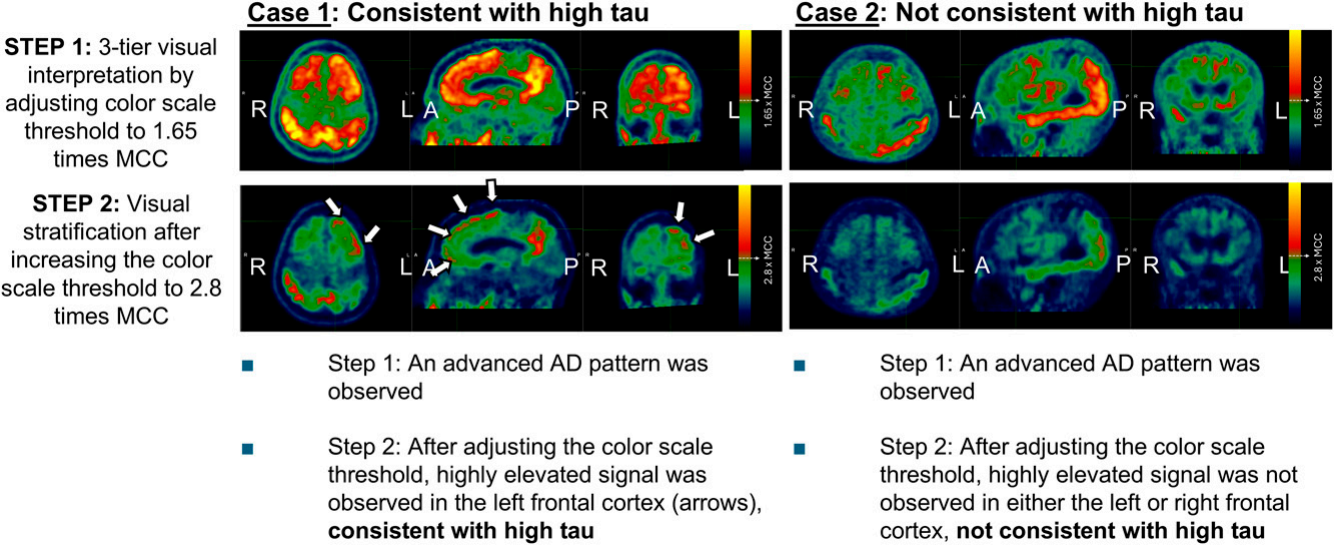
Two example cases of FTP visual stratifications. Scans that are consistent with advanced or moderate AD patterns using 3-tier visual interpretation (step 1) are further assessed in step 2. After color scale threshold adjustment, FTP scans with signal above threshold on either frontal lobe (arrows) are consistent with high tau (case 1), and others are considered not consistent with high tau. In Supplemental Figure 1, alternative color scale of same example cases is presented. MCC is captured by manually drawn 2-dimensional region of interest as described in Tauvid Food and Drug Administration label protocol (7).

An earlier observational clinical study of participants with AD found that those with high tau levels, as determined through quantitation, exhibited a visually elevated neocortical signal (1.65 times the MCC) in the frontal cortex (9,11). However, 40% of participants who also exhibited this elevated signal in the frontal cortex did not have high tau levels (12). This suggests that relying solely on visual assessment of elevated neocortical activity in the frontal lobe may have very high sensitivity but lacks the necessary specificity to identify quantitatively high tau. Therefore, it was hypothesized that increasing the visual read threshold of 1.65 × MCC and assessing the frontal lobe would improve the specificity and accuracy of identifying patients with high tau (Figs. 2 and 3). After proof-of-concept tests, a threshold of 2.80 times the MCC was identified to be optimal for identifying high tau (18). Further details on development of the visual read-based stratification method are given in the supplemental materials.

In study A26, FTP PET scans were evaluated by 5 independent readers using a 2-step approach (Fig. 2). First, scans were read using the 3-tier visual read method (9) and were labeled as either consistent with AD or not consistent with AD. In the second step, only the scans labeled as consistent with AD were further evaluated by readjusting the color scale, with the visual threshold for increased FTP signal being set to 2.80 × MCC. After readjustment of the color scale, 4 specific brain regions (temporal, occipital, parietal, and frontal) were bilaterally scored as either positive or negative based on the elevated signal above 2.80 × MCC, regardless of the signal intensity or extent (Fig. 3 and alternative color scale example in Supplemental Fig. 1). The FTP scans that were scored as positive on either frontal lobe (left or right) in the second step were stratified as visually consistent with high tau. All other FTP scans were stratified as visually not consistent with high tau. Scores captured from regions other than frontal (parietal, temporal, occipital) in step 2 were collected but not used or tested for stratification of high tau.

### Reader Training

Before conducting visual reads, 5 imaging physicians underwent computer-based training on the tau PET scan visual read method. This training consisted of explaining the steps of interpretation followed by a practice session with demonstration cases and a diverse set of practice cases. Demonstration and practice cases were selected from tau PET scans obtained from other internal clinical studies and did not include any tau PET scans from the TB2 trial. Once training was completed, each reader independently and blindly interpreted all scans without access to any clinical, demographic, or quantitation information.

### Endpoints and Statistical Analyses

The primary analysis for study A26 evaluated whether the visual stratification of FTP scans was associated with high-tau status, as determined using quantitative analysis of the same FTP scans. An AD-signature FTP SUVr of more than 1.46 was used as the reference standard of truth for the presence of high tau. The primary endpoints were positive percent agreement (PPA) and negative percent agreement (NPA) between high-tau visual read interpretation and high tau as determined by quantitation. The 95% CIs were estimated using the Wilson score method (21). Analyses were repeated for each of the 5 readers. Success criteria were met if the lower bounds of a 2-sided 95% CI for PPA and NPA were 50% or greater for at least 3 readers. PPA and NPA were calculated using the following formulas:

$$\text{PPA}=100\times \left(\text{TP}/\left(\text{TP}+\text{FN}\right)\right)\text{ and}$$

$$\text{NPA}=100\times \left(\text{TN}/\left(\text{TN}+\text{FP}\right)\right),$$

where TP is true-positive, TN is true-negative, FP is false-positive, and FN is false-negative.

The secondary analysis for this study evaluated the interreader reliability of FTP visual stratification (high tau versus non–high tau) across the 5 independent readers. Interreader reliability was assessed using a Fleiss κ-statistic. Success criteria were met if the Fleiss κ was at least 0.64 and the 95% CI lower bound was at least 0.55.

Two prespecified exploratory analyses were conducted. First, intrareader reliability was measured using Cohen κ for the test–retest scans (*n* = 20). Second, interreader reliability was measured using a Fleiss κ-statistic for the scans that failed quantitation at screening (*n* = 5). Fleiss κ- and Cohen κ-statistics were measured for visual stratifications based on parietal, occipital, and temporal regions as part of a post hoc analyses that had no implications for high-tau stratifications.

## RESULTS

### Clinical and Demographic Baseline Characteristics

Patient demographics and clinical characteristics between high-tau and non–high-tau groups were comparable, except for age, baseline amyloid PET burden, and tau PET SUVr (Table 1). Patients in the high-tau quantitation group were younger than those with non–high-tau quantitation (mean, 69.7 ± 6.6 y vs. 74.4 ± 5.7 y, *P* < 0.001 [Wilcoxon test]), had higher baseline centiloid levels (110.0 ± 31.3 vs. 97.3 ± 36.4, *P =* 0.007 [Wilcoxon test]), and had higher tau PET SUVr (mean, 1.71 ± 0.20 SUVr vs. 1.18 ± 0.12 SUVr; *P* < 0.004 [Wilcoxon test]). Additionally, the high-tau group had a slightly higher percentage of female patients (68.6% vs. 58.6%, χ^2^ = 1.51, *P =* 0.219 [Pearson χ^2^ test]); however, this difference was not statistically significant.

**TABLE 1.** Patient Demographics and Clinical Characteristics

Characteristic	Non–high-tau quantitation (SUVr ≤ 1.46, *n* = 70)	High-tau quantitation (SUVr > 1.46, *n* = 70)	Total (*n* = 140)	*P*
Age (y)	74.4 ± 5.7	69.7 ± 6.6	72.1 ± 6.6	*F*_1,138_ = 19.8, *P* < 0.001*
Male	29 (41.4%)	22 (31.4%)	51 (36.4%)	$\chi_{1}^{2}$ = 1.51, *P* = 0.219^†^
Race and ethnicity^‡^				
Asian	8 (11.4%)	1 (1.4%)	9 (6.4%)	$\chi_{2}^{2}$ = 5.8, *P* = 0.054^†^
White	61 (87.1%)	68 (97.1%)	129 (92.1%)	
Black or African American	1 (1.4%)	1 (1.4%)	2 (1.4%)	
Not Hispanic or Latino	66 (95.7%)	67 (97.1%)	133 (96.4%)	$\chi_{1}^{2}$ = 0.21, *P* = 0.649^†^
ApoE4 carrier	49 (70.0%)	47 (67.1%)	96 (68.6%)	$\chi_{1}^{2}$ = 0.13, *P* = 0.716^†^
Total MMSE score^§^	23.5 ± 3.4	20.5 ± 3.8	22.0 ± 3.9	*F*_1,136_ = 24.28, *P* < 0.001*
CDR global score^‖^				
0.5	45 (64.3%)	38 (54.3%)	83 (59.3%)	$\chi_{1}^{2}$ = 1.59, *P* = 0.451^†^
1.0	24 (34.3%)	30 (42.9%)	54 (38.6%)	
2.0	1 (1.4%)	2 (2.9%)	3 (2.1%)	
Flortaucipir PET				
τAD++	66 (94.3%)	69 (98.6%)	135 (96.4%)	$\chi_{1}^{2}$ = 1.87, *P* = 0.172^†^
AD-signature weighted neocortical SUVr	1.18 ± 0.12	1.71 ± 0.20	1.44 ± 0.31	*F*_1,124_ = 8.4, *P* = 0.004*
Florbetapir PET centiloid	97.3 ± 36.4	110.0 ± 31.3	103.7 ± 34.4	*F*_1,138_ = 7.46, *P* = 0.007*

* Wilcoxon test.

† Pearson test.

‡ Percentages are based on number of subjects with nonmissing data.

§ MMSE was missing for 2 participants (1 high-tau quantitation and 1 non–high-tau quantitation).

‖ CDR global score was missing for 3 participants (2 high-tau quantitation and 1 non–high-tau quantitation).

MMSE = mini mental state examination; CDR = clinical dementia rating.

Table does not include 5 randomly selected participants who failed quantitation at baseline or 20 τAD-negative scans that were used to avoid reader bias by reading only positive scans. Qualitative data are number and percentage; continuous data are mean ± SD.

### Visual Stratification Applied for High Tau

The median PPA and NPA across readers were 83.4% (ranging from 74.3% to 90.0%) and 88.9% (ranging from 87.1% to 91.4%), respectively, with lower bounds of 2-sided 95% CIs being at least 50% for all 5 readers (Table 2). Median overall percentage agreement (or accuracy) with quantitation-based stratification was 86.1% (ranging from 82.9% to 88.6%, Table 2). The Fleiss κ-point estimate was 0.8882 (95% CI, 0.8356–0.9409), and the Cohen κ overall point estimate was 0.9599 (95% CI, 0.9049–1.000), indicating almost perfect inter- and intrareader agreement.

**TABLE 2.** Agreement of High Tau Visual Read with Quantitation-Based High Tau (AD-Signature SUVr > 1.46)

Reader	PPA	NPA	Overall agreement
1	82.9% (72.4%–89.9%)	90.0% (80.8%–95.1%)	86.4% (79.8%–91.1%)
2	84.3% (74.0%–91.0%)	88.6% (79.0%–94.1%)	86.4% (79.8%–91.1%)
3	90.0% (80.8%–95.1%)	87.1% (77.3%–93.1%)	88.6% (82.2%–92.8%)
4	85.7% (75.7%–92.1%)	87.1% (77.3%–93.1%)	86.4% (79.8%–91.1%)
5	74.3% (63.0%–83.1%)	91.4% (82.5%–96.0%)	82.9% (75.8%–88.2%)
Overall	83.4% (79.2%–87.0%)	88.9% (85.1%–91.7%)	86.1% (83.4%–88.5%)

All physician readers conducted visual reads on same set of baseline FTP PET scans. Data in parentheses are 95% CIs estimated using Wilson score method (21).

**FIGURE 4.**
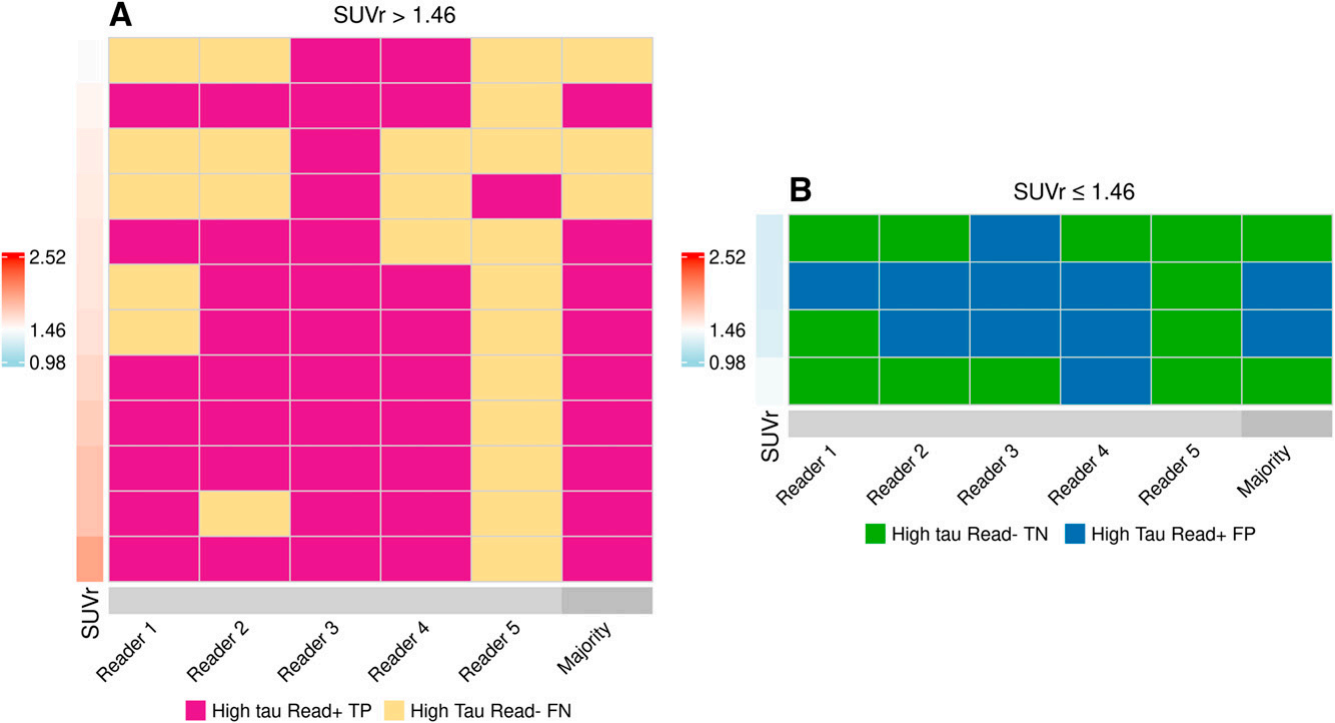
Tile plots of high-tau visual interpretations that were read in disagreement by at least 1 of 5 readers. (A) Tau PET scans that had high tau on quantitation. (B) Tau PET scans that did not have high tau on quantitation. Perfect agreement cases (not included in figure) consisted of 124 of 140 scans (88.6%). FN = false-negative; FP = false-positive; TN = true-negative; TP = true-positive.

Among 140 scans interpreted, 124 (88.6%) were assessed in perfect agreement among the 5 readers. Sixteen scans (11.4%) were read in disagreement by at least one of the readers (Fig. 4). Nine scans (6.4%) were assessed in agreement for 4 of the 5 readers. Twelve of the 16 scans were high tau (Fig. 4A) and 4 were non–high tau on quantitation (Fig. 4B). All individual readers had a Cohen κ of at least 0.89, and 3 of the 5 readers had perfect intrareader agreement (i.e., Cohen κ-point estimate of 1.000), indicating 100% test–retest reproducibility on the determination of high tau or non–high tau. For the set of 5 scans that failed eligibility quantitation, the Fleiss κ-point estimate was 0.7024 (95% CI, 0.6030–0.8018).

**FIGURE 5.**
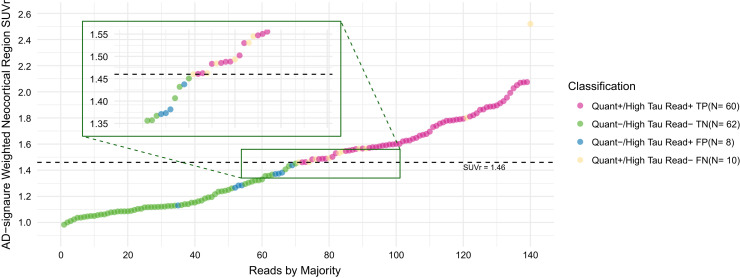
FTP PET quantitation and reader concordance graph. Dashed line represents high tau threshold of AD-signature weighted neocortical SUVr (1.46). Ten of 18 scans that were read as discordant with quantitation (FPs and FNs) had SUVr values relatively close to high tau cut point (SUVr, 1.46 ± 0.1), as represented in rectangle region. FN = false-negative; FP = false-positive; TN = true-negative; TP = true-positive.

Subjects who were stratified to high tau on the majority visual read had a significantly higher AD-signature SUVr than those who were stratified to non–high tau (1.67 ± 0.25 vs. 1.21 ± 0.29, Supplemental Fig. 2). Among tau PET scans that had high tau on quantitation, on majority read results, 60 and 10 scans were read as true-positive and false-negative, respectively (Supplemental Fig. 3). Likewise, among tau PET scans that had non–high tau on quantitation, 62 and 8 scans were read as true-negative and false-positive, respectively (Supplemental Fig. 2). Ten of 18 scans that were read discordant with the quantitation (false-positive and false-negative) had SUVr values relatively close to the high-tau cut point (SUVr, 1.46 ± 0.1; Fig. 5). The scans that were read as false-positive on the majority read had a mean SUVr of 1.32 ± 0.096, whereas the scans that were read as false-negative had a mean SUVr of 1.64 ± 0.323. Overall, the majority read-based assessment yielded a PPA of 85.7% (CI, 75.7%–92.1%) and an NPA of 88.6% (CI, 79.0%–94.1%).

### Post Hoc Analyses

The Fleiss κ-scores for the parietal, occipital, and temporal regions were 0.85, 0.66, and 0.79, respectively. The Cohen κ-statistics for the parietal, occipital, and temporal regions are presented in Supplemental Table 1.

## DISCUSSION

In this study, we presented results from an independent reader study (i.e., study A26) that tested a newly developed FTP visual read–based stratification method and its specific application for identifying patients with quantitation-based high tau (AD-signature SUVr > 1.46). Study A26 met all primary and secondary endpoints, demonstrating that the novel FTP visual read stratification method can help differentiate between early symptomatic AD subjects who have quantitation-based high tau and non–high tau burden. Among 5 imaging physicians who participated in the study, the overall PPA, NPA, and percent agreement for identification of high tau were high (83.4%, 88.9%, and 86.1%, respectively). Additionally, there was almost perfect agreement among readers (Fleiss κ, 0.89) and almost perfect agreement on repeated reads by individual readers (Cohen κ exceeds 0.89 for all readers).

Previous analyses have demonstrated that patients in the high-tau group had significantly worse cognitive decline than patients in other tau groups as assessed by the Mini-Mental Status Exam, Alzheimer Disease Assessment Scale, and Functional Activities Questionnaire (10). This observation was consistent with the evaluations in TB2, where participants with high baseline tau levels in the placebo arms had worse cognitive decline than did participants in the low- or medium-tau placebo groups (14). As such, identifying patients with a high tau burden in a clinical research setting could support AD staging and prognosis and advance scientific understanding in therapeutic and observational trials.

The visual read-based stratification method used in this study was applied to stratify FTP scans with high tau and required evidence of a highly elevated signal (2.80 times higher than MCC) in either the left or the right frontal lobe (Figs. 2 and 3). In a typical AD pathologic cascade, the frontal cortex is known to be one of the regions that accumulate tau at later stages of the disease, typically after the parietal cortex (22). The findings from study A26 were consistent with the expected progression of tau pathology in typical AD, indicating a high density of tau pathology in the frontal cortex, which reflects advanced global tau accumulation (9,11).

The developed FTP visual stratification method can be adapted to meet clinical and research stratification needs by focusing on different regions or combinations of regions and/or adapting the visual read significance threshold. A potential future application could be stratifying patients onto other tau PET groups such as low and medium tau using cortical regions other than frontal and alternative visual read thresholds. As part of the read method tested in study A26, readers were asked to also examine parietal, temporal, and occipital regions with the adjusted threshold of 2.80 times MCC. After a post hoc analysis, we observed that the inter- and intrareader agreements were also high between the reads of these regions, suggesting the potential feasibility of the method if, in the future, other cortical regions need to be used. We hypothesize that an adjusted threshold (either higher or lower) should yield similar inter- and intrareader agreement scores. Since study A26 was designed to evaluate the visual stratification method specifically for identifying high tau, future studies will be needed to validate stratification approaches for identifying other tau groups.

**FIGURE 6.**
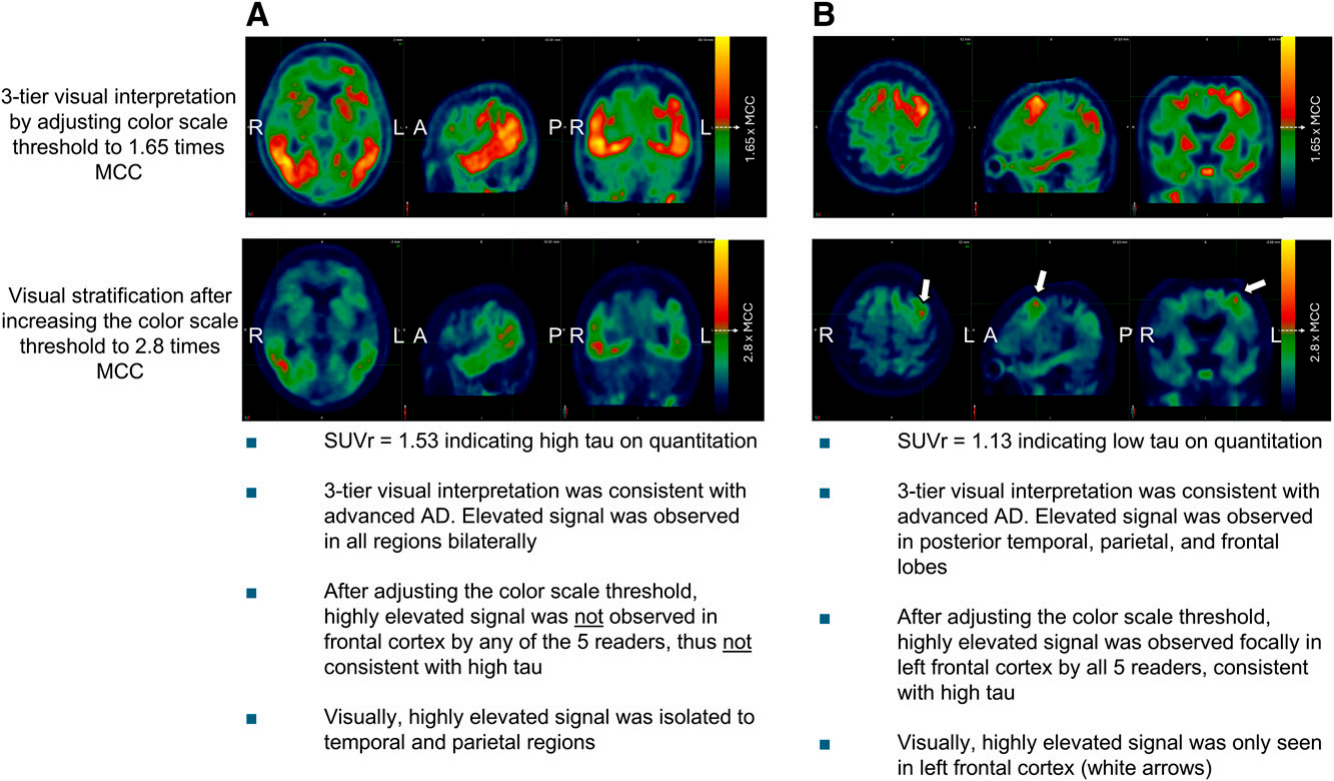
Two example cases that have discordance between visual stratification and quantitation. (A) Case with high tau on quantitation but not on visual stratification. (B) Case with low tau on quantitation and high tau on visual stratification. For both cases, all 5 readers had consistent visual read results. A = anterior; P = posterior.

There were several limitations of the study. First, data from study A26 showed almost perfect inter- and intrareader agreement, yet the accuracy results were not perfect (86.1% overall). Cases that had discordant reads (between readers and on test–retest) mostly consisted of borderline scans with focal elevation in the frontal cortex, which are more sensitive to the impact of manually captured MCC used to set the color scale (Supplemental Fig. 4). On the other hand, most of the discordant cases between visual and quantitative-based high tau determinations had quantitation levels (i.e., SUVr) close to the high-tau threshold (Fig. 5). We also acknowledge that the visual stratification method may not be able to fully capture at least some of the atypical tau PET patterns (e.g., high tau patients without highly elevated uptake on the frontal cortex, Fig. 6A; or non–high tau patients who have highly elevated uptake on the frontal cortex, Fig. 6B). However, using highly elevated frontal cortex uptake as the primary indicator of a high tau group provided simplicity for readers, helping achieve almost perfect intra- and interreliability without sacrificing much of the predictive ability. Additionally, in our previous proof-of-concept study (18), a visual read approach that focused on a global elevated signal, although optimized, still had lower accuracy in predicting high tau than did the frontal visual read approach (Supplemental Table 3).

Second, the 5 readers tested only a small random subset of 140 cases from TB2. However, study A26 was adequately powered, with over 90% statistical power to detect the primary endpoint. The random selection strategy ensured that the subset was representative of the trial population. Additionally, the readers underwent training using only an offline video session, and all reads were conducted independently and masked to demographic, clinical, and PET quantitation information, suggesting robust testing.

Third, the method and the thresholds were specific for FTP; without further testing, translating the methodology to other tau PET tracers would not be possible. Emerging tau PET harmonization efforts (i.e., CenTauR scale (23)) may have the potential to allow for better comparison between tracers and trials and mitigate the effects of this limitation in the near future.

Lastly, the method was tested on FTP images that were obtained from a strictly standardized and controlled imaging protocol as part of a clinical trial, TB2. As such, potential future applications in the real-world clinical setting might differ because of the intrinsic sensitivity of tracers to acquisition parameters and the addition of diverse populations that can potentially affect both quantitation and visual interpretations.

## CONCLUSION

The FTP visual stratification method consistently identified AD patients with a quantitation-derived high tau burden. Inter- and intrareader agreements were both high, indicating the reliability of this method. The visual stratification method was reliable for scans that had failed quantitation. This method does not require specialized software or image processing and can potentially be implemented using software available in clinical settings. Future investigations could potentially include expanding the validation dataset, including real-world clinical data with diverse populations, confirming through autopsy, further exploring alternative regions and thresholds for other tau PET stratifications, and, finally, assessing differences in treatment response to ATTs between visually high-tau patients and visually non–high-tau patients.

## DISCLOSURE

All authors are employees and potential shareholders of Eli Lilly and Company. No other potential conflict of interest relevant to this article was reported.

## Notes

KEY POINTS **QUESTION:** Could a novel FTP PET visual stratification method help with stratifying early symptomatic AD patients into different quantitation-based tau groups? **PERTINENT FINDINGS:** Study A26 met all primary endpoints and demonstrated that a FTP visual stratification method enables trained physician readers to differentiate between subjects with early symptomatic AD who have a quantitation-based high tau burden (AD-signature SUVr > 1.46) versus a non–high tau burden. The overall observed PPA, NPA, and percent agreement for identification of high tau were high (83.4%, 88.9%, and 86.1%, respectively). Additionally, there was almost perfect agreement among readers (Fleiss κ, 0.89) and within individual readers on test–retest reads (Cohen κ exceeds 0.89 for all readers). **IMPLICATIONS FOR PATIENT CARE:** The FTP PET visual stratification method does not require specialized software or image-processing steps and can potentially support AD staging and prognosis, also advancing scientific understanding in therapeutic and observational trials.
